# The genetic landscape and possible therapeutics of neurofibromatosis type 2

**DOI:** 10.1186/s12935-023-02940-8

**Published:** 2023-05-23

**Authors:** Mohammad Amin Ghalavand, Alimohamad Asghari, Mohammad Farhadi, Farzad Taghizadeh-Hesary, Masoud Garshasbi, Masoumeh Falah

**Affiliations:** 1 ENT and Head and Neck Research Center and Department, The Five Senses Health Institute, School of Medicine, Iran University of Medical Sciences, Tehran, Iran; 2 Department of Medical Genetics, Faculty of Medical Sciences, Tarbiat Modares University, Tehran, Iran; 3 Skull Base Research Center, The Five Senses Health Institute, Hazrat Rasoul Akram Hospital, Iran University of Medical Sciences, Tehran, Iran; 4 Radiation Oncology Department, Iran University of Medical Sciences, Tehran, Iran

**Keywords:** Neurofibromatosis type 2, NF2, Merlin, Meningiomas, Vestibular schwannoma, Acoustic neuroma, Hearing loss, Molecular targeted therapy

## Abstract

Neurofibromatosis type 2 (NF2) is a genetic condition marked by the development of multiple benign tumors in the nervous system. The most common tumors associated with NF2 are bilateral vestibular schwannoma, meningioma, and ependymoma. The clinical manifestations of NF2 depend on the site of involvement. Vestibular schwannoma can present with hearing loss, dizziness, and tinnitus, while spinal tumor leads to debilitating pain, muscle weakness, or paresthesias. Clinical diagnosis of NF2 is based on the Manchester criteria, which have been updated in the last decade. NF2 is caused by loss-of-function mutations in the *NF2* gene on chromosome 22, leading the merlin protein to malfunction. Over half of NF2 patients have de novo mutations, and half of this group are mosaic. NF2 can be managed by surgery, stereotactic radiosurgery, monoclonal antibody bevacizumab, and close observation. However, the nature of multiple tumors and the necessity of multiple surgeries over the lifetime, inoperable tumors like meningiomatosis with infiltration of the sinus or in the area of the lower cranial nerves, the complications caused by the operation, the malignancies induced by radiotherapy, and inefficiency of cytotoxic chemotherapy due to the benign nature of NF-related tumors have led a march toward exploring targeted therapies. Recent advances in genetics and molecular biology have allowed identifying and targeting of underlying pathways in the pathogenesis of NF2. In this review, we explain the clinicopathological characteristics of NF2, its genetic and molecular background, and the current knowledge and challenges of implementing genetics to develop efficient therapies*.*

## Introduction

**Fig. 1:**
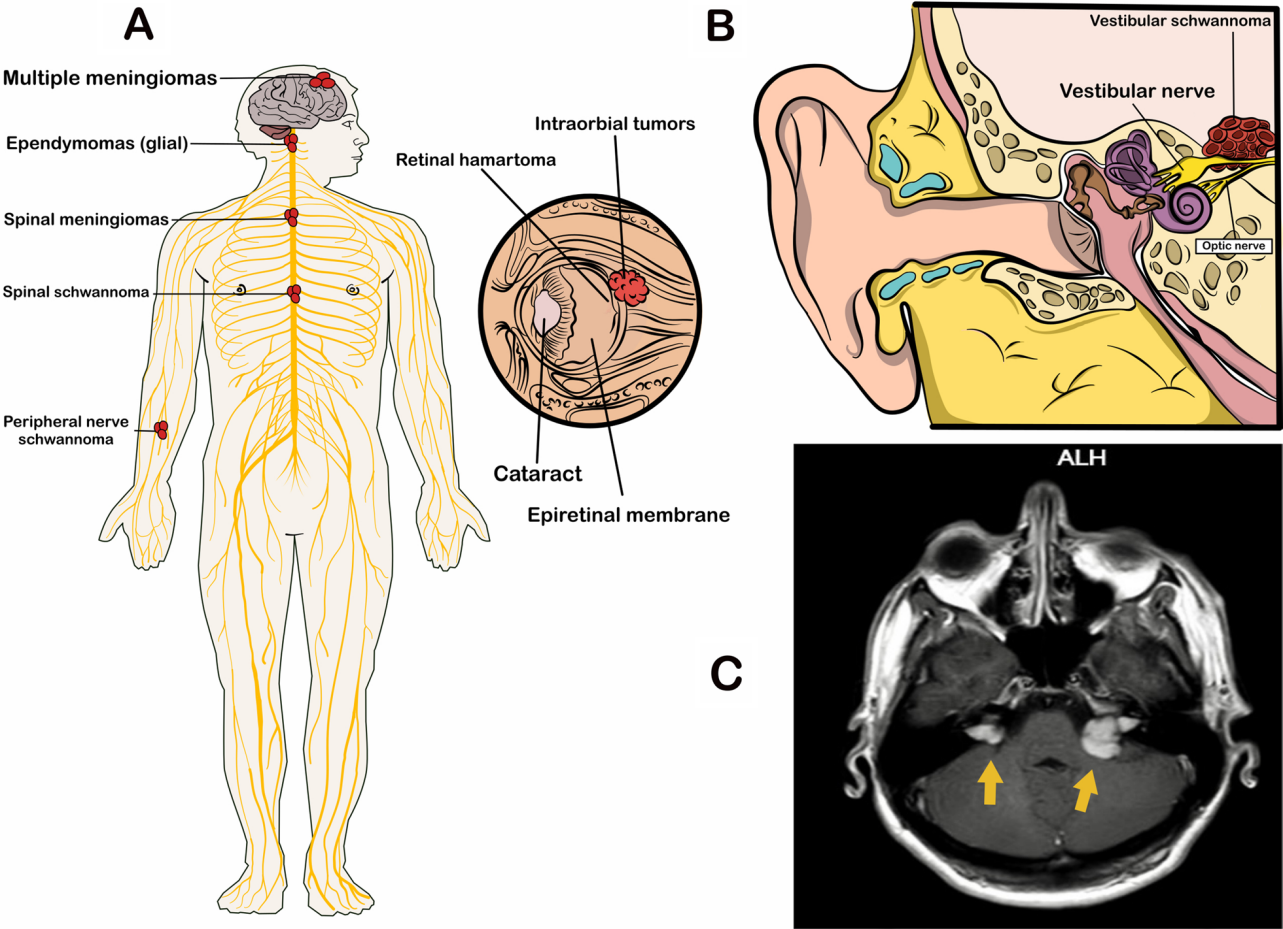
Schematic illustration and magnetic resonance imaging (MRI) of tumors associate with neurofibromatosis type 2 (NF2). **A** Different clinical symptoms associated with NF2. **B** A schematic depiction of vestibular schwannoma arising from eighth cranial nerve. **C** MRI of bilateral vestibular schwannoma as a hallmark feature of NF2 patients. The figure is redrawn from refs [2, 13].

Neurofibromatosis (NF) is a multiple tumor predisposing syndrome classified into: type 1 (NF1), type2 (NF2), and schwannomatosis [1]. NF1 is the most common type caused by mutations in the tumor suppressor *NF1* gene (OMIM: 613113) on chromosome 17. In comparison, schwannomatosis is caused by mutations of *SMARCB1* (SWI/SNF related, matrix associated, actin dependent regulator of chromatin, subfamily B, member 1) (OMIM: 601607) and *LZTR1* (Leucine zipper-like transcription regulator 1) (OMIM: 600574) genes on chromosome 22, encoding tumor suppressor proteins [1, 2]. The clinical description of NF2 was provided by Scottish surgeon JH Wishart in 1822 by dissecting a young male with multiple brain tumors originating from the skull [3]. NF2 is a mixed neuro-cutaneous genetic disease predisposing to the development of multiple benign tumors throughout the lifetime. The development of bilateral vestibular schwannoma (VS) (aka acoustic neuroma) is a characteristic of NF2. Hearing loss, tinnitus, and balance dysfunction are common symptoms of VS. Other common tumor characteristics include schwannomas of the cranial, spinal, and peripheral nerves as well as intracranial and intraspinal meningiomas (Fig. 1) [4].

The incidence of NF2 is around 1 in 25,000 [5, 6]. The prevalence of diagnosed patients is 1 in 100,000 dues to different reasons, such as lack of medical resources, insidious onset of clinical manifestations, and high rate of novel and somatic mutations [5–7]. Despite benign behavior, NF2-associated tumors result in considerable morbidity and mortality rates [2]. The early signs of NF2 usually manifest in the late teens or early twenties. The survival period after diagnosis is about 15 years, and the average age at death is between 36 and 39 years, with a 10-year survival rate of 67% [5, 8]. These rates illustrate the importance of NF2 management, despite its benign features.

Currently, there is no established approach to prevent or cure NF2 [1]. Cytotoxic chemotherapeutics are generally ineffective due to the benign biology of NF2-associated tumors [9]. NF2 can be managed by surgery, stereotactic radiosurgery, monoclonal antibody bevacizumab, and close observation. Even though these approaches can provide good local control, their shortcomings impede their general application. Despite all care, surgery can be associated with complications due to direct nerve manipulation or vascular events during the intervention. On the other hand, the concern due to radiation-induced secondary malignancy can enormously affect the patients’ quality of life. Moreover, the application of surgery and radiotherapy might be limited for tumors in the context of NF2 because the tumors are typically multiple in these patients [10, 11]. These concerns and shortcomings have led scholars to approach systemic therapies targeting NF2-associated tumors.

The advent of molecular biology to scheme the pathogenesis of NF2 can guide to address of new therapeutic potentials for the effective prevention and treatment of NF2-related tumors. NF2 is caused by loss of function mutations, deletions, or epigenetic modifications of the *NF2* gene (OMIM: 607379) [12]. The *NF2* gene encodes a 70-kDa protein called merlin, mainly acting as a tumor suppressor and participating in diverse cell signaling pathways [10]. Tumorigenesis occurs upon merlin loss of function by modifying downstream signaling pathways. Being such, the collaborative proteins of merlin can serve as novel targets for NF2 treatment [13–15].

Genotype–phenotype correlation and the role of NF2 genetic investigation in differentiating NF-related disorders highlight the importance of conducting genetic testing to detect at-risk patients, prognostication, and treatment [7, 16]. Also, it helps to find new treatments by reversing pathologic genetic/epigenetic modifications to replace normal merlin function, decrease tumor burden, preserve hearing, and improve survival of patients with NF2.

The present review focuses on the clinical features, diagnostic criteria, and the genetic and epigenetic background of NF2. It then highlights the current position and future perspectives of NF2 treatment.

## Genetic overview

Type 2 neurofibromatosis occurs due to the alterations of the *NF2* gene, located on chromosome 22q12.2 [17]. The defective gene is dominantly inherited and has nearly 100% penetrance by 60 years of age [16, 18]. The inactivation of the two alleles of the *NF2* gene is consistent with Knudson’s two-hit hypothesis. The *NF2* gene has 17 exons and encodes a 595-amino acid protein. The ultimate result of the translation of this gene is a protein called merlin, also known as neurofibromin 2 or schwannomin, which is a cell membrane-related protein with tumor suppressor activities [19].

### *NF2* gene variants

Nearly half of the patients with NF2 have de novo mutations without a family history, and about 60% of patients with novel mutations are mosaic [20]. This event is the result of post-zygotic interruptions during embryo development. Therefore, only a small population of cells will obtain the defective *NF2* gene [5]. This incident might challenge the detection of the mutations in peripheral blood analysis. In this condition, the molecular examination of tumor samples has more sensitivity [21]. Mosaic NF2 patients manifest milder to no symptoms depending on the nature of the mutations (Table 1) [22]. Typically, mosaic patients present with less severe hearing loss, fewer bilateral VS, CNS tumors, and the development of ocular signs at older ages. Patients with sporadic mosaic NF2 can exhibit clinical symptoms 7–8 years later than sporadic non-mosaic cases [23]. The risk of defective gene inheritance in mosaic patients is less than 50 percent; however, if inherited, offspring would develop more severe phenotypes in contrast with their parents because of a larger number of affected cells as a result of the transmission of the mutated gene to the next generation [24].

**Table 1:** Neurofibromatosis type 2 (NF2) mutations based on location and their clinical manifestations [16, 26]

Mutation Type	Location	Mosaic^†^ mutation	Germline mutation
Truncating mutation	Exon 1	Moderate	Moderate-Severe
	Exon 2–13	Moderate-Severe	Severe
	Exon 14–15	Moderate	Moderate-Severe
Splice site mutation	Exon 1–7 (in frame)	Mild	Moderate
	Exon 1–7 (frameshift)	Moderate	Moderate-Severe
	Exon 8–13 (in frame)	Mild	Moderate
	Exon 8–13 (frameshift)	Moderate	Moderate-Severe
	Exon 14–17	Mild	Moderate
Large and small deletions	Small in-frame deletion or duplication	Very mild phenotype	Mild
	Large deletion (> 1 exon) including promoter or exon 1		
	Maintaining reading frame	Very mild phenotype	Mild
	causing frameshift alteration	Mild	Moderate
	Whole NF2 gene	Mild	Moderate
	Large deletion (> 1 exon) excluding promoter or exon 1		
	Maintaining reading frame	Very mild phenotype	Mild
	Causing frameshift alteration	Mild	Moderate
Missense variants		Very mild phenotype	Mild

^†^NF2 gene pathogenic variant in an unaffected tissue such as blood saliva samples with variant allele frequency < 50%

The type and severity of NF2 manifestations depend on the type of gene mutation. The variability within families is usually fewer than variabilities between families, implying a considerable influence of the underlying genotype. Evidence favors a substantial link between genotype and phenotype for NF2-related disorders [16, 25]. The types of pathogenic mutations can predict the number of intracranial meningiomas, spinal tumors, and tumors of peripheral nerves [7]. The UK NF2 Genetic Severity Score was developed—per clinical manifestations and genotypes—to categorize the patients based on severity into severe, moderate, and mild. [16] These groups are different in age at diagnosis and show different manifestations. The UK NF2 Genetic Severity Score has recently been validated in the Spanish NF2 cohort (Table 1) [26].

### Genotype–phenotype correlation in NF2

Generally, NF2 due to truncating mutations (nonsense or frameshift) is more severe and appears at younger ages [27]. Evidence denotes that patients with truncating mutations are more likely to develop symptoms earlier (before 20 years), have a greater risk of developing at least two CNS tumors in addition to VS (before 30 years), and have a shorter average life expectancy [16]. Missense mutations and large deletions usually give rise to milder phenotypes. However, the underlying mechanisms linking large deletions and milder NF2 phenotypes are still unknown [27]. Unlike truncating and missense mutations, splice site mutations are associated with more diverse phenotypes (Table 1) [28].

The involved site also matters. Mutations in the amino-terminal domain of NF2 proteins are associated with early tumor onset with more severe disease progression [21]. It has been shown that exons 2 and 3 are necessary for merlin’s self-association, which is required for its tumor suppressor activity. In mouse models, the knockout of this part of the gene gave rise to higher tumorigenesis in Schwan cells [29, 30]. Patients with truncating variants of the 3’ region of exons 2 through 13 have more severe presentations with poor survival outcomes than the same variants involving exons 1, 14, and 15. Moreover, frameshift variants close to the *NF2* translation initiation codon improve life expectancy [16, 28]. Re‐evaluation of missense variant classifications indicated that most *NF2*‐associated variants are located in exon 7, and minor variants are toward the C‐terminus of the NF2 protein [31].

Mutations at the amino-terminal domain of the NF2 are associated with more meningiomas, [32] especially in the intracranial space [33]. The Wishart phenotype, also known as the severe form, is associated with truncating mutations and alterations at the amino-terminal domain of merlin [34, 35]. In contrast, the Gardner phenotype, also known as the adult form of NF2, is associated with missense or splice site mutations, especially at the carboxy-terminal. Generally, the Gardner phenotype has a better prognosis with a lower risk of meningioma [32].

## Epigenetic overview

Epigenetics, defined as heritable alterations in gene expression that do not result in permanent changes in DNA sequence, plays a crucial role in maintaining cell identity and phenotypic characteristics [36] Through epigenetics, cells can undergo differentiation and development into various cell lines. The main epigenetic mechanisms include DNA modifications, chromatin modifications, and non-coding RNA interactions. DNA modification, especially methylation, occurs at CpG islands of gene promoters [37].

Research on monozygotic twins with NF2 introduced epigenetic changes as one of the main factors in phenotypic heterogeneity [38]. Further research indicated the epigenetic modifications at the 5’ flanking region of the *NF2* promoter [39]. Hyper-methylation in CpG islands leads to gene silencing by decreasing the mRNA expression of the *NF2* gene [40, 41]. Later, several studies reported a low level of methylation in the promoter region of the *NF2* gene in patients with sporadic VS. Therefore, *NF2* methylation may not serve as a primary reason for developing VS. [42, 43] A methylation-specific PCR on schwannomas has shown aberrant methylation in tumor-related genes including *THBS1*, *TP73*, *MGMT*, and *TIMP3*. [41, 44] Lassaletta et al*.* indicated aberrant methylation status in twelve tumor-related genes of patients with VS, including *RASSF1A*, *VHL*, *PTEN*, *TP16*, *CASP8*, *TIMP3*, *MGMT*, *DAPK*, *THBS1*, *HMLH1*, *TP73*, and *GSTP1*. Among these genes, the *RASSF1A* methylation is inversely correlated with the clinical growth index, and methylation in *CASP8* is associated with the patient's age and tumor size. The methylation of *TP73* is associated with hearing loss. [45] *TP73* is a tumor suppressor gene, mediating apoptosis in neural cells. However, the mechanism underlying its contribution to VS formation is yet to be determined. [46] Previous studies demonstrated the link between methylation of homeobox genes (*HOX*) and several malignancies, including leukemia and breast cancer. [47, 48] Genome-wide methylation analysis in VS demonstrated global hypomethylation at the *HOX* gene cluster [49]. Other epigenetic modifications pertaining to VS formation are post-transcriptional changes of *NF2*, alterations in lysine acetylation, and dysregulation of miRNA expression [50–53]. In meningiomas, epigenetics has been applied to categorize the subtypes. In fact, the DNA methylation-based classification and grading system of meningiomas has improved the prediction of tumor prognosis and recurrence by selecting clinically homogenous groups [54]. In the case of VS, however, this application is still in its infancy and requires further investigations. More research is required to delineate the genetic and epigenetic changes explaining the molecular and phenotypic differences between individuals with NF2.

## Clinical features

Tumors in the context of NF2 can involve central and peripheral nervous systems (Fig. 1a) [2]. The clinical manifestations of NF2 are in a wide range, from no symptoms to life-threatening symptoms, depending on the involved nerves [2]. The bilateral VS is the hallmark feature of NF2 that originate from myelin-forming Schwann cells in the vestibulocochlear nerve (Fig. 1b, c) [13]. VS is the main reason for hearing impairment, balance dysfunction, and tinnitus in NF2 patients. VS growth can compress the adjacent facial nerve, leading to facial weakness, numbness, or paresis. In advanced cases, life-threatening intracranial difficulties (e.g., hydrocephalus) might happen upon brain stem or cerebellar compression.

At least two-thirds of patients with NF2 might develop spinal tumors presented as debilitating pain, muscle weakness, or paresthesias [55, 56]. The most common NF2-related spinal tumors are schwannomas. These arise from the dorsal root and can take on a characteristic dumbbell shape. Most persons with spinal cord involvement have multiple tumors [57].

Approximately half of NF2 cases have meningiomas that usually present as multiple meningiomas with considerable morbidity due to seizures, paralysis, and headaches [2]. The incidence of meningiomas increases with age, and lifetime risk may approach 80% [32]. Most cases are intracranial, although intradural and extramedullary spinal meningiomas are also reported. Orbital meningiomas can lead to visual loss by compressing the optic nerve. Those at the skull base may cause cranial neuropathy, brain stem compression, and hydrocephalus [57]. As such, the site of involvement determines the severity and type of symptoms of NF2-related meningioma.

Individuals with NF2 may develop visual impairment due to cataract, optic nerve meningiomas, retinal hamartomas, and the epiretinal membrane [5]. Cataracts are reported in 60–80 percent of patients and typically are manifested as posterior subcapsular lenticular opacities. Lens opacities may appear prior to the onset of symptoms of VS and can be seen in children [58]. It has been demonstrated that ophthalmic manifestations can get worse with an increased genetic severity score. Painter et al*.* demonstrated that the prevalence of cataracts, optic atrophy, epiretinal membranes, and combined hamartomas significantly increased with genetic severity score. The authors also found that greater genetic severity is associated with greater visual morbidity at an earlier age [35]. These findings reflect the positive correlation between genetic mutations and clinical manifestations in NF2.

The cutaneous manifestations of NF2 are diverse, including plaque-like lesions (usually pigmented with hair overgrowth), subcutaneous nodules (often palpable along the peripheral nerves), and intracutaneous tumors. The great majority of these tumors are schwannomas [5]. Cutaneous involvement of NF2 may precede neurological and ophthalmic symptoms by several years, thereby can contribute to the early diagnosis [59].

Neurogenic manifestations of NF2 are various [5, 60]. A recognized feature of NF2 is mononeuropathy, particularly in childhood [61], which usually involves the facial nerve and can precede the development of other NF2 manifestations. It also can present as foot or hand drop. A progressive polyneuropathy of adulthood not directly related to tumor masses is also recognized [62].

VS presents in 90% of patients with NF2, usually manifested as a progressive hearing impairment [2, 25]. The definite pathophysiology of hearing loss in patients with VS is yet to be determined [13]. One possible mechanism is through mechanical pressure of the growing tumor inside the bony space of the auditory canal. Six studies found an association between VS tumor size and the severity of hearing impairment [63].However, this hypothesis is challenged by the finding that there is no consistent correlation between tumor size and the severity of hearing loss [64]. Furthermore, Sakamoto et al. found no significant correlation between hearing loss speed and tumor size [65]. In support, Caye-Thomasen et al. found that gradual or sudden hearing loss may occur without a change in tumor size or configuration [66]. Hence, the association between VS tumor size and the severity of hearing impairment remains an open question. Another hypothesis pertains to cochlear ischemia due to the compressive effect of the growing tumor on the supplying vessels [67]. Despite clinical evidence for this hypothesis [68, 69], it may not be generalized to all patients because the histological vascular changes were detected in a subset of patients. An emerging hypothesis has considered tumor-secreting molecules, including ototoxic and neurotoxic agents, as the leading cause of cochlear damage [70]. The extracellular vesicles secreted by tumor cells (so-called exosomes) can mediate cochlear damage [71]. Whether these mechanisms are in-whole or in-part justify VS-associated hearing loss is still unclear and requires further dedicated studies.

## Diagnosis

Over the past four decades, the diagnostic criteria of NF2 have been developed. Table 2 represents the previous and current diagnostic criteria for NF2. In 1987, the National Institutes of Health (NIH) published the first set of criteria [72]. Later, in 1992, the diagnostic criteria of NF2 were updated according to the Manchester consensus [73]. The NIH and Manchester criteria are merely based on the clinical features and significant family history. The diagnosis of NF2 is often postponed due to a wide clinical heterogeneity, particularly in cases without a significant family history or those with distinct manifestations prior to the VS development [74]. The advent of sequencing technologies has provided many opportunities to diagnose NF2 patients, especially for differential diagnosis and the mosaic form of NF2. In the 2016 revision of the Manchester criteria, the following updates were considered: patients with unilateral VS and nondermal schwannomas require testing for LZTR1 to rule out the schwannomatosis. In addition, the age limit of 70 years was considered for the development of VS, given the observation that up to 50% of individuals aged 70 and older may have bilateral VS without underlying mosaic or constitutional *NF2* mutation [33]. In 2019, the consensus updated the diagnostic criteria as follows: “glioma” and “neurofibroma” were removed from the NF-associated lesions, and “ependymoma” was added to the list [75]. In addition, the siblings were not considered as the “first-degree relative” in the criteria because of the zero positive predictive value [75]. However, these criteria were misleading for patients with multiple schwannomas. In the recent update (2022 consensus), the role of genetic testing was highlighted to discriminate NF2 from schwannomatosis. In this criteria, the “NF2” term was updated to “NF2-related schwannomatosis”, and the previous “schwannomatosis” became updated per the relevant pathogenic variant: SMARCB1-related schwannomatosis, LZTR1-related schwannomatosis, 22q-related schwannomatosis, schwannomatosis-NOS (not otherwise specified), or schwannomatosis NEC (not elsewhere classified) [76]. Overall, during the past four decades, the diagnostic criteria of NF2 have evolved from purely clinical to clinical-genetic criteria.

**Table 2:** Previous and current diagnostic criteria for neurofibromatosis 2

NIH criteria^a^	Manchester criteria^a^	2016 consensus^a^	2019 consensus^a^	2022 consensus^a^
1. Bilateral 8th nerve masses^b^	1. Bilateral VS	1. Bilateral VS aged < 70 years	1. Same as 2016	1. Bilateral VS
2. FDR with NF2, plus - unilateral 8^th^ nerve mass, or -two NF-related lesions^c^	2. FDR with NF2, plus - unilateral VS, or -two NF-related lesions^c^	2. FDR with NF2, plus unilateral VS aged < 70 years	2. FDR other than siblings with NF2, plus unilateral VS aged < 70 years	2. Identical NF2 pathogenic variant in ≥ two distinct NF2-related tumors^g, h^
	3. Unilateral VS, plus -two NF-related lesions^c^	3. FDR with NF2, or unilateral VS, plus two NF-related lesions^d, e^	3. FDR other than siblings with NF2, or unilateral VS, plus two NF-related lesions^f, e^	3. Two major or one major and two minor criteria, as follows:
	4. Multiple meningioma, plus -unilateral VS, or -two NF-related lesions^c^	4. Multiple meningioma, plus two of unilateral VS or other NF-related lesions^d^	4. Multiple meningioma, plus two of unilateral VS or other NF-related lesions^f, e^	Major criteria: -Unilateral VS -FDR other than siblings with NF2 - ≥ 2 meningiomas - NF2 pathogenic variant in an unaffected tissue (e.g., blood, saliva)
		5. Constitutional pathogenic NF2 gene variant in blood or identical mutations in two distinct tumors	5. Same as 2016	Minor criteria: - > one NF-related lesion - Juvenile subcapsular or cortical cataract, retinal hamartoma, epiretinal membrane in a person aged < 40 years -Specific pattern of genetic changes^i^

CT scan, computed tomography scan; FDR, first-degree relative; LTZR1, leucine zipper like transcription regulator 1; MRI, magnetic resonance imaging; NF2, neurofibromatosis type 2; NIH; national institute of health; VS, vestibular schwannoma

^a^The diagnosis is established if any criteria is met

^b^Based on CT scan or MRI

^c^Neurofibroma, meningioma, glioma, schwannoma, and juvenile posterior subcapsular lenticular opacity

^d^Neurofibroma, meningioma, glioma, schwannoma, cerebral calcification, cataract

^e^If unilateral VS and ≥ 2 non-intradermal schwannomas must be LZTR1 negative

^f^Meningioma, ependymoma, schwannoma, cerebral calcification, cataract

^g^Schwannoma, meningioma, and/or ependymoma

^h^If the variant allele fraction in unaffected tissues such as blood is clearly < 50%, the diagnosis is mosaic NF2

^i^According to ref. no [76]

The current diagnostic criteria for NF2 are based on clinical examinations and genetic tests. Genetic testing is suggested in all patients with suspected schwannomatosis predisposition syndromes [77]. The clinical assessments include family history, physical examinations such as cutaneous, ear, and eye examinations, and a contrast-enhanced MRI of the brain and whole spine [5]. Next-generation sequencing (NGS) offers an endorsed method to detect the *NF2* gene variation, with a detection rate of up to 90 percent for patients within familial NF2. However, the sensitivity of NGS for sporadic NF2 decreases to 25–60 percent because of somatic mosaicism [78]. NGS is more sensitive than Sanger sequencing at detecting genetic variants present at a low-level allele fraction (< 50%) [79]. Along with NGS, multiplex ligation-dependent probes and high-resolution karyotyping complete the genetic analysis of patients [76]. In the first step, investigating *NF2*, *SMARCB1*, and *LZTR1* in blood or saliva samples is recommended to detect the genetic background of patients with NF2 and the differential diagnosis. Thereafter, analysis of two independent tumor samples helps to detect second hit mutations. Finally, tumor tissue analysis for methylation patterns is suggested. New emerging evidence indicates the role of second hit mutation, epigenetic factor, and modifier genes in phenotypic variability within NF2 families [25, 76, 78].

Prognostic information, earlier intervention, and efficacious therapies are the robust benefits of early genetic testing in patients with suspected NF2-related diseases [2]. Moreover, understanding the genomic and molecular pathogenesis of *NF2* variants will provide insights into better knowledge about tumorigenesis-related manifestations, such as meningioma, spinal schwannomas, ependymomas, and dermal schwannomas [25]. In addition, it can improve diagnostic precision to better discriminate NF2-related schwannomatosis from its differential diagnosis [76]. Finally, pre-and post-test genetic counseling allows patients and their relatives to make informed decisions and improves management.

## Treatment

There is currently no cure for NF2, so management focuses on close observation and treating problems when they arise. The National Health Service recommends active monitoring for asymptomatic cases with annual brain MRI to find or follow the brain tumors, annual eye tests, and annual hearing tests [80].The significant impacts of NF2 on the patients’ quality of life and the severity of symptoms urge the need to explore effective therapies. Therefore, treatment recommendations for NF2-related tumors aim to preserve of the physiologic function and quality of life [5]. Hence, incidental identification of a tumor is not an indication for treatment per se, and the potential benefits must be compared against the risks of active intervention. Treatment is usually selected when there is a risk of brainstem compression, hearing impairment, or facial nerve dysfunction. The management of patients with NF2 is typically determined in a multidisciplinary setting, consisting of neurotologists, audiologists, neurologists, neurosurgeons, oncologists, ophthalmologists, and geneticists [2].

VS are usually managed surgically if treatment is indicated. Besides, first-line bevacizumab (a humanized anti-VEGF monoclonal antibody) therapy has indicated promising results in rapidly growing tumors. Currently, however, there is no FDA-approved targeted therapy for NF2 [13]. Bevacizumab has shown promise in the treatment of progressive VS in NF2 patients [27]. A meta-analysis of 161 patients with NF2-related VS (total of 196 VS tumors) reported that bevacizumab resulted in partial regression, stable disease, and progression in 41% (95% CI 31–51%), 47%, (95% CI 39–55%), and 7% (95% CI 1–15%), respectively. Hearing improvement was reported in 20% (95% CI 9–33%), stability in 69% (95% CI 51–85%), and additional loss in 6% (95% CI 1–15%) [81].

Similarly, the mainstay treatment of progressive meningiomas threatening functional loss is surgery. Regarding inoperable cases, radiotherapy is also used. Most meningiomas grow to a specific size and stop; therefore, they do not require special treatment [82]. Bevacizumab is indicated to repress tumor growth in recurrent WHO grades II/III meningiomas [83].

Usually, there is no need for intervention in NF2-related ependymomas due to their slow growth rate. Occasionally, where intervention is needed, surgery is recommended [84]. Emerging evidence suggests that bevacizumab can improve the symptoms of NF2-associated ependymomas [85, 86].

Surgical interventions can also be applied to improve hearing impairments due to NF2 tumors. To this end, cochlear or auditory brainstem implants are extensively applied worldwide [87, 88].

The multifocal nature of NF2-related tumors, their proximity to the vital structures (e.g., brain stem and internal carotid artery), or neural involvement (e.g., facial and auditory nerves) can limit surgical interventions. In patients with NF2, radiotherapy can increase the risk of developing additional benign tumors in the irradiated field and malignant transformation of existing benign tumors [89]. Therefore, it is only applied in inoperable cases. In addition, there is still a risk of tumor recurrence after surgery or radiotherapy, with a long-term tumor relapse rate of 40% [1, 90–93]. Unfortunately, cytotoxic chemotherapies have limited efficacy in these patients due to the benign nature of NF-related tumors [94]. Since merlin plays a role in various cellular pathways involved in cell growth, proliferation, and also cell–cell interactions, identifying signaling pathways and major mediators of NF2 pathogenesis can provide new therapeutic perspectives in the treatment of NF2-related tumors.

To summarize, the treatment of NF2 must start with one critical question: how much do the treatment benefits outweigh its risks? This concern originates from two points: (a) long-term quiescence of NF2-related tumors [95] and (b) significant adverse effects of treatments (neural damages in surgical interventions and risk of secondary malignancy due to radiotherapy). The multifocality of NF2-related tumors and their proximity to critical structures can further limit the administration of surgery and radiotherapy in these patients. As such, systemic treatments can overpass these limitations. However, the available systemic therapies are limited to a handful of choices (e.g., VEGF or mTOR inhibitors) with limited efficacy. As noted, NF2 is a genetic disease. Hence, exploring its genetic and epigenetic backgrounds and the involved signaling pathways can help to find better treatments. The following sections discuss the role, structure, and function of merlin in the pathology and treatment of NF2.

## Molecular biology of merlin

Merlin is a member of the ERM protein family, and its name stands for ezrin-radixin-moesin-like protein. The ERM family members are highly conserved during the evolution, reflecting their crucial roles in human cells [96]. Generally, ERM family members provide cross-links between the plasma membrane and actin-based cytoskeleton, essential for plasma membrane maintenance and function. Nearly all ERM proteins have high similarities in their structure; however, slight differences result in different cell function [96].

Merlin binds to the actin cytoskeleton and is involved in the stabilization of the membrane cytoskeletal interface by interacting with PI3K (Phosphoinositide-3 kinase)/AKT (Ak strain transforming), Raf (Rapidly accelerated fibrosarcoma)/MEK (Mitogen-activated protein kinase)/ERK (Extracellular-signal-regulated kinase), Wnt/β-catenin, RTKs (Receptor tyrosine kinases), mTOR (Mechanistic target of rapamycin), and Hippo signaling pathway [97]. The interaction of merlin with the aforementioned biomarkers has another face. It has been demonstrated that merlin (as a tumor suppressor) has inhibitory effects on PI3K [98], Raf/ERK [99], Wnt/β-catenin [100], RTKs [1], and mTOR [101]. Hence, in patients with NF2, the inhibitory effect of merlin on these pathways is removed, and these pro-tumorigenic pathways become activated. This interaction can serve as an opportunity to design targeted therapies inhibiting the activated pathways in patients with NF2 (discussed in "Future therapeutic perspective" Section).

### Merlin structure

The *NF2* gene expresses ten different isoforms resulting from alternative splicing, among which isoforms I and II are the major ones and both have tumor suppressive function [102, 103]. Each isoform has a specific expression pattern throughout the body. Merlin is highly expressed during the embryo’s development, and specific isoforms (7 and 9) are absent in adult tissues. Generally, merlin can be primarily detected in neurons, Schwann cells, meningeal cells, and lens cells [104].

**Fig. 2:**
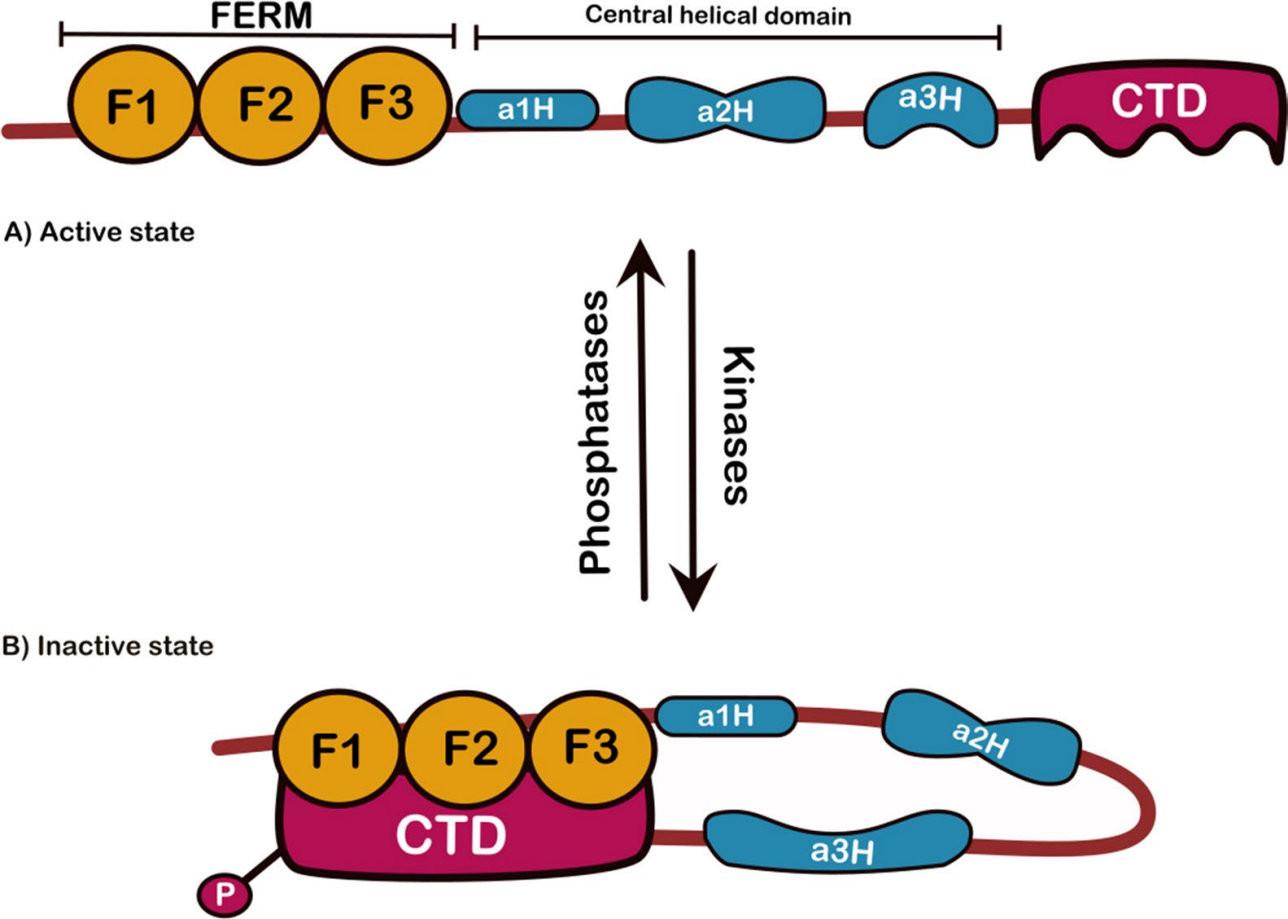
A schematic depiction of protein domain structure and states merlin. **A** Merlin is a multidomain protein and consists of three parts. The first part is at the N-terminus, which is highly conserved and is called FERM or N-terminal ERM association domain. The FERM itself has three subdomains F1, F2, and F3. The second part is the α- helical domain, which consists of three α- helices (α1H, α2H, and α3H). The third part is a short, mainly helical C- terminus domain (CTD) or C-terminal ERM association domain. **B** Merlin’s head-to-tail folding between FERM and CTD domains in monomer structure renders the protein in closed inactive conformation upon phosphorylation of Ser 518

**Fig. 3:**
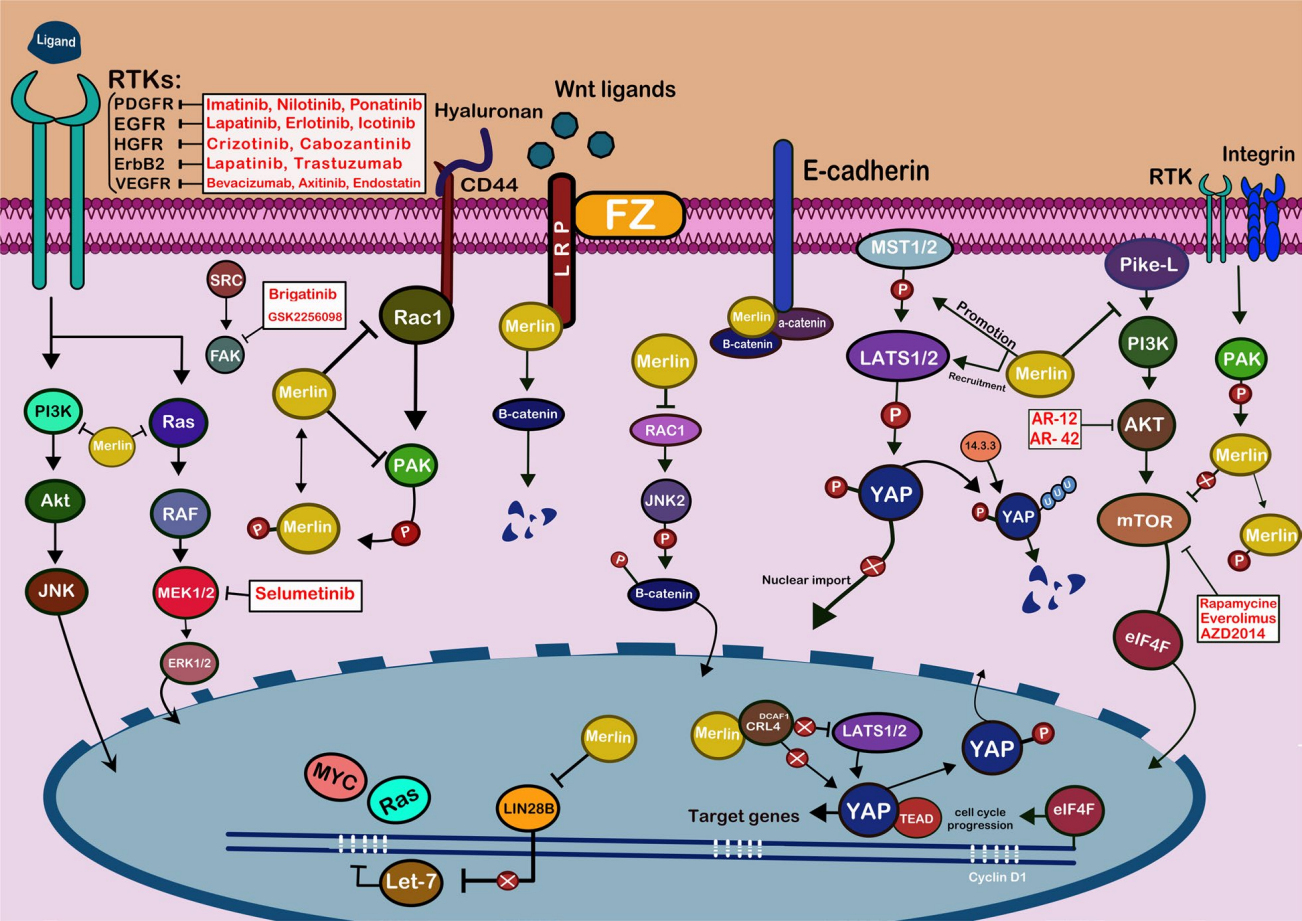
Merlin signaling pathways and potential therapeutic targets in NF2. A schematic depiction of the main intracellular pathways regulated by the protein product of the NF2 gene (merlin (is shown by golden)). Merlin regulates cell survival, proliferation, and cell–cell interaction in response to multiple proliferative signaling pathways at the plasma membrane and in the nucleus. Merlin can predominantly block the RTKs’ activity on their downstream targets, including RAS, PI3K, and Rac. Merlin inhibits Wnt/β-catenin signaling through inhibit translocation of β-catenin to the nucleus. At the nucleus (inhibition CRL4^DCAF1^) and cell cortex (promoting MST1/2), merlin can regulate Hippo signaling pathway. As a result, the expression of target genes of YAP will decrease. Merlin may also block LIN28B in the nucleus, reducing let-7 miRNA cluster repression and downregulating proto-oncogenic proteins including MYC and RAS. Diverse treatment options have been investigated for NF2 patients, including the suppression of merlin-regulated proteins and other cellular receptors. Pathogenic mutations in NF2 patients cause merlin function loss, which modulates downstream activity in each pathway, promoting cell growth, protein and fatty acid synthesis, proliferation, and survival. The five white boxes provided are current inhibitors of this pathway with their respective targets. Proteins are also illustrated in circular shapes, and each of them has been given a distinct color. the bilayer cellular plasma membrane with phospholipid compounds is presented at the top in pink. Also, the blue area in the cell represents the nucleus and the two blue straight lines within, represent genes involved in this pathway. Black arrows indicate the act of promotion and blocking lines indicate the act of suppression

Merlin is a multidomain protein consisting of three parts (Fig. 2). The first part is at the N-terminus, which is highly conserved in ERM family members and is called FERM (band 4.1, ezrin, radixin, moesin) or N-terminal ERM association domain. FERM binds to the plasma membrane or plasma membrane proteins, including adherens junctions and cell surface receptors like integrins, RTKs, CD44, CD43, ICAMs, and scaffolding/effector proteins [105, 106]. The FERM itself has three subdomains (F1, F2, and F3). These subdomains form a tri-lobed, cloverleaf conformation. The stabilization of this structure lies within the biochemical properties of regions between each subdomain residing on this domain. The second part of merlin is the α-helical domain, consisting of three α-helices (α1H, α2H, and α3H) and a hinge between the latter two. The α-helical domains α2H and α3H form a coiled-coil conformation in the monomer state [106]. The third part of merlin is a short, mainly helical C-terminus domain (CTD) or C-terminal ERM-association domain, which has specific biological functions and structure in each ERM family member. The CTD part regulates protein–protein or membrane-protein interactions and enables interactions with the FERM domain. The CTD contains specific residues that are targets for critical post translational modifications (PTM) [107]. Merlin (and other ERM family members) links the cell membrane and the basal actin cytoskeleton and mediates membrane remodeling, membrane structure maintenance, and vesicle trafficking [2]. Merlin may produce distinct cellular effects depending on its conformation and modifications. The significant difference is that ERM proteins can bind to the actin cytoskeleton via their actin-binding domain located on their C-terminus; however, in merlin, the actin-binding domain is located in the FERM domain (N-terminus) [108, 109]. In addition, merlin is the only member of the ERM family manifesting tumor suppressor activity [110]. Besides, merlin may have intranuclear effects. Its NLS (nuclear localization sequence) addresses its role in signaling pathways regulating gene expression (Fig. 3) [111].

### The ‘open’ and ‘closed’ states

The function of ERM proteins is regulated by transitioning between the ‘open’ and ‘closed’ states, leading to activation and deactivation of the protein, respectively. Merlin and other ERM proteins can form a hetero- or homodimer conformation by constructing a head (being the FERM domain)-to-tail (being the CTD) conformation at intermolecular and intramolecular levels [112]. During FERM-CTD interactions, the CTD (shaped as a mono-layer surface) covers the hydrophobic parts of the F2 and F3 subdomains within the FERM domain (Fig. 2) [113]. This interaction is highly conserved in the ERM protein family, including merlin. In the open state, merlin can interact with other substances, including plasma membrane or integral proteins, and act as a scaffolding protein mediating the signaling cascades [114]. Deactivation occurs when the protein is in the closed state, and some residues are not accessible for other modifications or interactions; exceptionally, the closed state of merlin triggers its tumor suppressor activity [115]. The factors involved in transitioning between the two states and the outcome of their interactions require more investigation. It has been proposed that the binding of phosphatidylinositol 4,5-bisphosphate and the PTM on specific residues can induce the open form [116]. The open form provides sites on FERM and CTD domains to bind the partner proteins. For example, the FERM domain can bind to proteins residing on the membrane, including CD44, CD43, PSGL-1, MT1-MMP, ICAM-1, ICAM-3, and neprilysin, as well as proteins involved in the Hippo signaling [117–119].

### Post translational modifications

Merlin has a multitasking feature since it functions both at the plasma membrane and in the nucleus (Fig. 3) [1]. This multitasking attribute results from merlin’s PTM by phosphorylation, cysteine modifications, ubiquitination, acetylation, or SUMOylation, resulting in structural and biochemical changes determining open or closed states (Fig. 2) [120, 121]. Most PTM occurs after the phosphorylation of specific residues of merlin domains, including Ser10, Ser13, Ser518, and Thr581. Ser10 and Ser13 are located in the merlin N-terminal domain. Ser10 shares a kinase partner with Ser518 and is phosphorylated by protein kinase A (PKA) in vivo. The phosphorylation of Ser10 is necessary for cell migration and regulating the morphology of the actin cytoskeleton [122].

Among these residues, Ser518 is of great interest. Ser518 residue is mainly located at the CTD. The phosphorylation of Ser518 residue can render the strength of interaction between FERM and CTD and mediates the formation of filopodia (the actin-rich protrusions from the cell surface sensing, migration, and cell–cell interaction) [123]. Ser518 is the target of PKA and PAK (P21 activated kinase) and results in the repression of merlin’s tumor suppressor activity and loss of contact inhibition [124]. Inactivation of merlin’s tumor suppressor activity can be inverted by an enzyme called myosin phosphatase MYPT1-PP1δ that dephosphorylates Ser518 residue (Fig. 2) [125].

Merlin concentration is regulated by AKT (also called protein kinase B) phosphorylation of residues Ser10, Ser315, and Thr230 leading to ubiquitin-mediated protein degradation. As a result, merlin cannot interact with its binding partners [126]. The exact consequences of these modifications and transitioning between the two states must be explored.

### Merlin in signaling pathways

Merlin regulates cell survival and proliferation in response to multiple proliferative signaling pathways (Fig. 3). As a result, the inactivation of merlin can lead to uncontrolled cell proliferation and tumorigenesis, especially in the nervous system. The mechanisms underlying how merlin regulates cell proliferation are yet to be elucidated. Recent evidence demonstrated the role of merlin in various pathways, including mTOR, RTKs, Wnt/β-catenin, PI3K/AKT, and Hippo pathways [10, 13].

RTKs, as a member of the protein tyrosine kinases family, are a group of membrane receptors that dimerize after ligand binding. The phosphorylation of RTK’s intracellular domain activates cell proliferation, survival, and migration signaling pathways [127]. Merlin is essential for blocking RTK signaling pathways [1]. For instance, merlin binds to CD44 and restricts it from binding to hyaluronan, an extracellular matrix component, and inducing contact growth inhibition by blocking the CD44-Rac axis (Fig. 3) [110, 128]. Merlin interacts with RTKs, such as PDGFR (Platelet-derived growth factor receptor), EGFR (Epidermal growth factor receptor), HGFR (Hepatocyte growth factor receptor), VEGFR (Vascular endothelial growth factor receptor), and ErbB2/ErbB3 via its FERM domain. Merlin can predominantly block the RTKs’ activity on their downstream targets, including RAS, PI3K, and Rac (Fig. 3) [10, 13]. In patients with VS, EGFR expression level is correlated with increased tumor size and younger age onset [129]. In addition, the expression and activation of PDGFR-α and PDGFR-β are increased in VS compared with intact nerves [130].

Compared with healthy individuals, the PI3K/AKT/mTOR pathway is enhanced in patients with VS. [131] PIKE-L (Phosphatidylinositol 3-kinase enhancer-L), a human GTP (guanosine triphosphate)-binding protein, activates the PI3K and increases cell proliferation through PI3K/AKT pathway. Merlin regulates this pathway by inhibiting PIKE-L, and loss of merlin induces tumorigenesis in schwannoma and meningioma by activating PI3K/AKT pathway and increasing cell proliferation [132, 133]. The mTORC1 signaling is highly activated in NF2-deficient mesothelioma, schwannomas and meningiomas, thereby inducing tumor growth and rapamycin sensitivity [101]. Loss of merlin results in an integrin-associated activation of mTORC1 signaling via PAK1 and increases the expression of cyclin D1 to promote cell cycle progression (Fig. 3) [134].

At the nucleus and cell cortex, merlin can regulate the Hippo signaling pathway. The Hippo pathway is essential for regulating cell survival and proliferation [135]. In the cell cortex, merlin acts as an upstream effector where it binds to Lats1/2 (Large tumor suppressor kinases) via its FERM domain and elevates their concentration at the plasma membrane. This interaction results in the activation of Lats1/2 proteins leading to further inactivation of YAP (Yes-associated protein) and transcriptional coactivator with PDZ-binding motif (TAZ) [136]. Phosphorylation of YAP/TAZ is conducive to its degradation or cytoplasmic retention by binding to the 14.3.3 protein complex residing in the cytoplasm. With the absence of merlin, YAP/TAZ will be stabilized within the nucleus and bind to their protein partner TEAD (TEA domain transcription factor) and in turn, promote tumor cell survival and proliferation [136]. In patients with schwannoma, the expression of YAP and its transcription targets, including PDGFRβ, HER2, and HER3, are significantly elevated [137]. In the nucleus, unphosphorylated merlin binds to CRL4^DCAF1^(DDB1- and Cul4-Associated Factor 1), an E3 ubiquitin ligase, thereby inhibiting its role in YAP activation [97]. Moreover, binding to CRL4^DCAF1^ blocks the LATS1/2 degradation, which in turn increases YAP phosphorylation and its inactivation (Fig. 3) [138].

Wnt/β-catenin pathway counts as another target for merlin. Usually, Wnt signaling increases the nucleus translocation of β-catenin, enhances the expression of c-Myc and cyclin D1, and increases cell proliferation by TCF/LEF (T-cell factor/lymphoid enhancer factor) transcription factors [139]. The merlin connection to LRP6 (Low-density lipoprotein receptor-related proteins 6) by the FERM domain centralizes the β-catenin in the cytoplasm [100]. In addition, merlin in a complex with β-catenin, α-catenin, and E-cadherin localizes β-catenin near the plasma membrane [140]. Alternatively, merlin can prevent the transfer of β-catenin into the nucleus by inactivating Rac1 (Ras-related C3 botulinum toxin substrate 1). Usually, phosphorylated β-catenin by Rac1-activated JNK2 (c-Jun N-terminal kinase) is translocated to the nucleus (Fig. 3). [141].

The RNA-binding protein called Lin28B (Lin-28 homolog B), which regulates cellular growth and reprogramming, is another partner of merlin. Lin28B can suppress the generation of Let-7 (Lethal-7), a miRNA with a tumor suppressor activity that inhibits the expression of various proto-oncogenes, including MYC and RAS. Merlin can interact with Lin28B via its FERM domain, allowing an intact generation of Let-7 through Lin28B sequestration in the cytoplasm (Fig. 3) [142].

## Future therapeutic perspective

A comprehensive understanding of molecular mechanisms in tumor progression of NF2-related tumors will provide opportunities for exploring more efficient treatments. Currently, many drugs are being examined targeting pathways involved in the pathogenesis of NF2 [14, 94, 143]. In this section, clinical trials evaluating the targeted therapies for NF-related tumors are discussed (Fig. 3, Table 3).

**Table 3:** Completed and ongoing clinical trials on patients with neurofibromatosis type 2

ID	Initiation Date	Phase	Nation	N	Disease	Treatment	Primary Outcome	Class of inhibitor	Mechanism of action	Status
NCT02934256	7.2016	2	China	10	NF2 VS	Icotinib	Volume of tumor	RTKs	EGFR inhibitor	Completed
NCT02129647	4.2014	2	USA	13	NF2 VS	Axitinib	Expression Levels of p-S6,p-ERK, p-AKT	RTKs	VEGFR1/2/3 inhibitor	Completed Has Results
NCT02104323	1.2014	2	China	20	NF2 VS	Endostatin	Volume of tumor	RTKs	VEGF expression inhibitor	Completed
NCT01345136	7.2015	2	USA	4	NF2	Everolimus	VS volume	mTOR	Inhibits mTORC1	Active, not recruiting
NCT01490476	1.2012	2	France	10	NF2	Everolimus	VS volume	mTOR	Inhibits mTORC1	Completed
NCT01880749	6.2013	1	USA	5	NF2 VS Meningioma	Everolimus	Proportions of VS and meningiomas after exposure	mTOR	Inhibits mTORC1	Completed
NCT01419639	10.2011	2	USA	10	NF2	Everolimus	Radiographic Response Change in Tumor Size	mTOR	Inhibits mTORC1	Completed, has results
NCT01767792	5.2013	2	USA	22	NF2 Progressive VS	Bevacizumab	Hearing	RTKs	VEGF-A inhibitor	Completed Has Results
NCT01207687	10.2010	2	USA	14	NF2 VS	Bevacizumab	Hearing	RTKs	VEGF-A inhibitor	Completed Has Results
NCT01125046	6.2010	2	USA	50	Acoustic Schwannoma Meningioma Ependymoma NF1 NF2 Brain Tumor	Bevacizumab	Progression Free Survival	RTKs	VEGF-A inhibitor	Completed Has Results
NCT04283669	2.2020	2	USA	19	NF2 Progressive VS	Crizotinib	Volumetric response rate	RTKs	c-MET inhibitor	Active, not recruiting
NCT02831257	8.2016	2	USA	18	NF2 Meningioma	AZD2014	Radiographic Response Rate	mTOR	mTOR kinase inhibitor	Completed Has Results
NCT03071874	10.2017	2	USA	28	Grade II/ III NF2-mutated meningiomas	AZD2014	Progression Free Survival	mTOR	mTOR kinase inhibitor	Active, not recruiting
NCT04374305	6.2020	2	USA	80	NF2 VS	Brigatinib	Volumetric response rate	RTKs	ALK and EGFR inhibitor	Recruiting
NCT02523014	8.2015	2	USA	124	NF2 Gene Mutation	GSK2256098	Progression free survival	FAK	FAK inhibitor	Recruiting
NCT03095248	5.2017	2	USA	34	NF2 VS Meningioma Ependymoma Glioma	Selumetinib	Hearing Response rate of NF2 related tumors	MEK	MEK1/2 inhibitor	Recruiting
NCT03079999	6.2018	2	USA	300	NF2 VS	Aspirin	Progressive free survival	Hippo pathway	Cyclooxygenase Inhibitor	Recruiting
NCT01129193	1.2017	1	USA	44	Solid tumors	AR-42	Adverse events	AKT	Histone deacetylase inhibitor	Completed
NCT02282917	12.2015	1	USA	7	NF2 VS Meningioma	AR-42	Ratio of Phospho-AKT	AKT	Histone deacetylase inhibitor	Terminated Has Results
NCT05130866	6.2022	2/3	USA	89	NF2	AR-42	Progressive free survival	AKT	Histone deacetylase inhibitor	Recruiting
NCT00863122	6.2009	1	USA	26	NF2 VS Auditory Tumor	Lapatinib	Lapatinib Plasma Concentrations	RTKs	EGFR/ ErbB2 Inhibitor	Completed Has Results
NCT00973739	9.2009	2	USA	21	NF2 VS	Lapatinib	Progression Free Survival	RTKs	EGFR/ ErbB2 Inhibitor	Completed Has Results
NCT01201538	10.2010	2	Canada	2	Growing VS	Nilotinib	Volume change of VS	RTKs	Bcr-Abl inhibitor	Terminated
NCT00911248	7.2009	2	USA	11	NF2	PTC299	Volume of tumor	RTKs	VEGFA inhibitor	Terminated
NCT04085159	9.2019	1/2	China	100	Schwannomatosis NF1 NF2	Antigen-specific T cells CART/CTL and DCvac	Percentage of adverse effects	Immunotherapy	Immunotherapy	Recruiting
NCT05228015	7.2022	1	USA	158	NF2 Deficiency	IK-930	Safety and tolerability	Hippo pathway	TEAD inhibitor	Recruiting
NCT04857372	10.2021	1	USA	156	Advanced Mesothelioma and Other Solid Tumors	IAG933	Percentage of adverse effects	Hippo pathway	YAP/TEAD interaction inhibitor	Recruiting

NF2: neurofibromatosis type 2; RTKs: receptor tyrosine kinases; EGFR: epidermal growth factor receptor; VS: vestibular schwannoma; VEGFR: vascular endothelial growth factor receptor; mTORC1: mechanistic target of rapamycin complex 1; cMET: c-mesenchymal-epithelial transition; ALK: anaplastic lymphoma kinase; MEK; Mitogen-activated protein kinase; AKT: Ak strain transforming

### Targeting the PI3K/AKT/mTORC1 pathway

The PI3K/AKT/mTORC1 pathway serves as a hub in the intracellular signaling hierarchy by integrating signals from various upstream pathways and regulating cell proliferation, metabolism, and apoptosis [144]. As mentioned in Sect. 7.4, merlin can suppress the constitutively active mTORC1 effector. Therefore, mTORC1 can serve as a target for VS treatment [101]. In vivo evidence has shown an efficacy of a mTORC1 inhibitor (sirolimus). In this study on a mouse xenograft model, sirolimus (aka rapamycin) induced growth arrest in the growing VS tumors [145]. Everolimus, a rapamycin analog with an mTOR kinase inhibitor activity, has been evaluated in different clinical trial studies [146, 147]. In a phase II clinical trial on patients with VS in the context of NF2 (NCT01419639), 10 mg/day of everolimus on continuous daily dosing for 12 months resulted in stable disease in five out of nine patients (55.5%) and progressive disease in the remaining four (45.5%). No high-grade toxicities were recorded. Although no clinical response was reported, the significant disease control rate was considered encouraging for further studies [147]. Four-year follow-up of patients revealed that one patient remained with stable disease. In this study, three patients with progressive disease on everolimus were treated with bevacizumab. One out of three patients had stable disease after 8-month treatment. This study indicated that the progressive disease on everolimus may not preclude further treatment with bevacizumab [146].

Two VS features are slightly elevated *AKT* gene expression and marked AKT protein phosphorylation, which is necessary for proper AKT activity [15] AKT phosphorylation is primarily mediated by phosphoinositide-dependent kinase-1 (PDK1). An in vivo study demonstrated that AR-12 (OSU-03012), a PDK1 inhibitor, can proceed schwannoma cells to apoptosis by inhibiting AKT phosphorylation in VS and malignant schwannoma cells [15, 148]. Another study revealed that a novel histone deacetylase inhibitor, AR-42 (OSU-HDAC42), inhibits the downstream *AKT* and *PDK1* expression, leading to G2 arrest and apoptosis in VS cells [149]. Two pilot studies of AR-42 in human NF2, VS, and meningiomas (NCT01129193 and NCT02282917) were safe and well tolerated, and no grade 3–4 toxicities were seen at the low dose 40 mg (pilot 2) three times weekly for three weeks of a 28-day cycle [150, 151]. A phase II/III randomized trial on 89 patients with NF2 or NF2-mutated recurrent meningiomas 12 years and older is recruiting (NCT05130866).

### Targeting RTKs

The importance of different RTKs (including VEGFR, EGFR, PDGFR, and ErbB2/3) in the pathogenesis of NF2 has been established. Given the inhibitory effects of merlin on RTKs activity, several studies have examined the RTK inhibitors in patients with NF2.

#### VEGF-A/VEGFRs

VEGFRs activation stimulates the signaling cascade of angiogenesis upon binding to its substrate, VEGF. The expression of VEGF and VEGFR-1 is related to VS's growth rate, leading to an increased vessel density and abnormal cellular proliferation [152]. In a murine VS xenograft model, the inhibition of VEGF inhibited tumor growth (mean: 50%) and improved survival (more than 50%) [153].

Bevacizumab (an anti-VEGF antibody) has demonstrated encouraging results in patients with NF2-associated VS. [13, 14] In patients with progressive disease, it has successfully controlled the growth rate and helped to improve the hearing status [81, 154–156]. Different studies indicated a 36%–41% partial response of bevacizumab for NF2-related VS in patients 12 years and older [14]. The therapeutic effect of bevacizumab in pediatrics and adults with smaller and slow-growing tumors was less prominent [156–158]. The median treatment duration was 16 months, which does not produce a long-lasting effect. It led to severe toxicity in 17% (95% CI 10–26%), including amenorrhea, proteinuria, and hypertension [81]. The bevacizumab side effects are dose-dependent. A retrospective study reported 62% proteinuria and 58% hypertension in patients with NF2 treated with a 5 mg/kg, biweekly regimen [159]. In comparison, another study demonstrated that dose reduction to 2.5 mg/kg was associated with stable disease in all patients without significant side effects [160]. New evidence indicated hearing improvement and tumor volume reduction in safety and preliminary efficacy of the VEGFRs peptide vaccine in patients with progressive NF2 [161]. The DCE-MRI (Dynamic contrast-enhanced magnetic resonance imaging) findings showed that bevacizumab could improve vascular perfusion and oxygen supply by restoring the normal function of tumor vasculature. This function decreased tumor edema and improved the radiation effect in an NF2 schwannoma model [162]. It has been demonstrated that treatment of progressive meningioma (after surgery and radiotherapy) with bevacizumab led to disease stabilization with 6-month progression-free survival rates of 87, 77, and 46% in grades I to III meningiomas [83]. Studies on other inhibitors of VEGF (endostatin) and VEGFR (axitinib) did not demonstrate encouraging results [14, 163].

The VEGF level circulating in the peripheral blood can serve as a predictive biomarker to identify NF2 patients who benefit from anti-VEGF therapy [13]. The evidence showed that increased HGF level is a poor prognostic factor of hearing after bevacizumab therapy [154, 164]. This finding addresses that HGF/cMET (C-mesenchymal-epithelial transition) signaling pathway may underly the hearing loss in patients with VS and determines the response to bevacizumab [165].

#### HGFR

HGFR (c-MET) is an RTK participating in cancer progression [166] and (chemo) radiotherapy resistance [167]. Both HGF and HGFR are overexpressed in sporadic VS. [168] Several studies have evaluated the effects of combination RTK inhibitors in NF2-related VS cell lines. An in vitro study demonstrated that targeting VEGF-A reduced c-MET expression and targeting c-MET reduced VEGF-A expression. This finding suggests a crosstalk between c-MET and VEGFA in VS biology [165]. Therefore, the combination of RTK inhibitors is a potential treatment for NF2-related VS. An in vivo study indicated that crizotinib, a c-MET inhibitor, can improve radiosensitivity in NF2 schwannoma cells. It is demonstrated that low-dose radiation concurrent with crizotinib was as effective as high-dose radiation. Therefore, concurrent crizotinib can help to improve hearing with reduced radiation doses and fewer toxicities [169]. A phase II clinical trial of crizotinib for children and adults with NF2 and progressive VS is ongoing (NCT04283669). In an in vitro study on merlin-deficient mouse schwannoma cells, the combination therapy with cabozantinib (c-MET inhibitor) and saracatinib (Src inhibitor) suppressed the growth of the cells and promoted caspase-dependent apoptosis [170].

#### ErbB

The ErbB family is a group of RTKs consisting of four members, including ErbB1 (EGFR/HER1), ErbB2 (HER2/neu), ErbB3 (HER3), and ErB4 (HER4). Activation of ErbB receptors requires dimerization upon binding to the ligand. All four members of the ErbB family can form heterodimers. It has been evidenced that ErbB family members participate in Schwann cell differentiation and proliferation [171]. Therefore, they can serve as targets to halt VS growth in patients with NF2. EGFR and ErbB2 heterodimers are the most common ErbB receptor dimerization type reported in VS. [172] Lapatinib has a dual inhibitory impact on EGFR/ErbB2 [129]. A phase II trial on NF2 patients with progressive VS indicated that lapatinib resulted in improved hearing in 4/13 patients (30.7%) and radiographic response in 4/17 patients (23.5%) [173]. It has been demonstrated that lapatinib continuation has the potential to arrest or reduce the growth of NF2-related meningiomas [174]. A phase II study on the efficacy and safety of icotinib, an oral EGFR, indicated minimal toxicity and also radiographic and hearing responses in patients with NF2 and progressive VS. [175] In a nude mouse model, both erlotinib (an EGFR inhibitor) and trastuzumab (a HER2/neu inhibitor) significantly inhibited the development of VS xenografts [176]. However, a clinical study on eleven patients with NF2-related VS demonstrated poor hearing and radiographic responses with erlotinib (150 mg daily) [177].

#### PDGFR

Imatinib mesylate inhibits PDGFRs and their downstream signaling pathways in VS cells. This action enhances apoptosis and decreases cell viability in a dose-dependent manner [178]. In addition, imatinib can prevent angiogenesis in both sporadic and NF2-related VS. The dual inhibitory effect of imatinib on tumorigenesis and angiogenesis has made it a promising drug for further trials on schwannoma [179]. Nilotinib, a second-generation RTK inhibitor, has a similar mechanism of action and structure to imatinib, with greater lipophilicity. This feature improves its tissue penetration with lower toxicity and higher efficacy [180]. The suppression of PDGFRs and their downstream signaling mediators (AKT and mTOR) can justify their anti-tumorigenic action [180]. Ponatinib has been gaining more attention for its potential in treating VS. In merlin-deficient human Schwann cells, ponatinib promotes G1 arrest by inhibiting the phosphorylation of PDGFRα/β, AKT, MEK1/2, ERK1/2, STAT3, and p70S6K [181].

Combination therapy with a dual mTORC1/2 kinase inhibitor vistusertib (AZD2014) and dasatinib (a multi-kinase inhibitor) demonstrated encouraging results as a novel therapeutic strategy for VS. [182] Notably, the synergic effect of AZD2014 and dasatinib results in fewer toxicities because lower doses can be administered. Moreover, the clinical trial (NCT03071874) has administered vistusertib to treat recurrent grade II or III NF2-mutated meningiomas to inhibit the mTORC1/C2 complexes. [14].

### Targeting hippo pathway

The Hippo pathway, an evolutionarily conserved mechanism that controls tissue homeostasis, is one of the most well-known merlin-regulated pathways [136]. Multiple research projects have been conducted to examine the impact of targeting the Hippo pathway on NF2-related tumors. For instance, YAP knockdown halted tumorigenesis initiation and induced a decrease in cell proliferation of NF2-deficient meningioma and mesothelioma cell lines [183–185].

YAP1/TAZ's prooncogenic abilities are assumed to be mediated by TEADs (discussed in Sect. 7.4), thus disrupting their interaction can be a therapeutic target. Therefore, many compounds have been developed and assessed to produce novel anti-cancer drugs related to this interaction. The first small compound demonstrated to impede YAP-TEAD binding was verteporfin, a photosensitizer utilized therapeutically in photodynamic treatment for neovascular macular degeneration [186]. This small molecule has been shown to inhibit different forms of carcinomas such as hepatoma and glioblastoma and retinoblastoma [187]. In addition, it was demonstrated that verteporfin inhibits YAP activity as well as the viability, invasion, and tumor sphere formation of mesothelioma cell lines [188]. IAG933, another inhibitor of the interaction YAP-TEAD, is ongoing in a multi-center phase I clinical trial on patients with mesothelioma, NF2 mutated tumors, and tumors with functional YAP/TAZ fusions (NCT04857372). Furthermore, IK-930, a small molecule inhibitor of TEAD, is undergoing a phase I clinical trial on adult patients with advanced or metastatic solid tumors (NCT05228015). IK-930 prevents palmitate binding and thereby interrupts improper TEAD-dependent transcription [189].

Mammalian vestigial-like 4 (VGLL4) has previously been discovered as a natural YAP antagonist that binds to TEADs via its Tondu (TDU) domain and the VGLL4 TDU region is sufficient to block YAP activity. Jiao et al*.* indicated that disruption of YAP-TEADs interaction by a VGLL4-mimicking peptide may be a promising therapeutic strategy for YAP-driven human cancers [190].

Hippo signaling has been associated with the cellular metabolic state, including cellular responses to glucose restriction (energy stress) and the mevalonate cascade [191]. Glucose shortage lowers cellular ATP levels and activates AMP-dependent protein kinase (AMPK), a sensor of cellular energy stress. AMPK has been reported to decrease YAP activity through a range of processes, including reducing nuclear YAP levels and YAP-TEAD interactions, which provide new avenues to target this pathway more efficiently [192]. A recent study evaluated the metabolic aspect of YAP/TAZ-depleted in NF2-deficient schwannoma [193]. This study indicated that YAP/TAZ depletion reduces glycolysis-dependent growth and elevates mitochondrial respiration and reactive oxygen species buildup, resulting in oxidative stress-induced cell death. Moreover, they showed lysosome-mediated cAMP-PKA/EPAC-dependent activation of RAF-MEK-ERK signaling as a resistance mechanism to YAP/TAZ inhibition [193].

### Targeting other pathways

Selumetinib, a MEK inhibitor, is the first FDA approved drug for NF1-associated plexiform neurofibromas in 2020. It showed 66% response rate for inoperable or progressive plexiform neurofibromas in children two years and older with a duration of response more than one year in 82% [194]. Phase II clinical trial of selumetinib for NF2-related tumors is underway (NCT03095248) [14].

Brigatinib, an anaplastic lymphoma kinase (ALK) inhibitor, is a potential choice for NF2-related tumors. It has been demonstrated that brigatinib causes tumor shrinkage in both NF2-deficient meningioma and schwannoma by inhibiting multiple tyrosine kinases, including EphA2, Fer, and focal adhesion kinase 1 (FAK1). Brigatinib can also inhibit multiple RTKs frequently activated in these tumors but not ALK [195]. Brigatinib is under investigation in an undergoing phase II clinical trial involving 80 patients (NCT04374305).

Another FAK inhibitor, GSK2256098, was evaluated in recurrent or progressive grade I-III meningiomas as part of the first genomically driven phase II trial. Patients with *NF2* mutations were treated with GSK2256098 (750 mg orally twice daily) until progressive disease. It was well tolerated and improved progression-free survival at 6 months [196].

In summary, if we put the clinical studies of VS into account, evidence on bevacizumab is more than other targeted therapies. It could provide 88% clinical benefit (response rate 41%) out of 196 VS tumors [81]. Bevacizumab can also help to alleviate the compression effects on critical structures (by reducing edema) and can be applied as a radiosensitizer by enhancing tissue oxygenation [162]. In the second place is everolimus (a mTORC inhibitor) with a clinical benefit of 55% (all as stable diseases) [147]. This option makes bevacizumab available for progressive cases (clinical benefit rate 33%) [146]. Another available choice for VS is lapatinib (an ErbB2 inhibitor). It has improved hearing in 30% of cases with progressive VS with a clinical response of 23% [173].Lapatinib can also be applied in NF2-related meningioma [174]. The following sections present emerging and potential treatment choices based on the other aspects of NF2 pathophysiology.

### Targeting proinflammatory mediators

The growing evidence emphasizes the importance of the tumor microenvironment (TME) in the progression and development of VS pathology, and inflammation counts as the most important factor in tumors' growth [197]. B and T lymphocytes and macrophages, especially a particular type of macrophage called tumor-associated macrophages (TAMs), are among the most important immune cell infiltrating the VS tissue [198–200].TAMs originate from circulating bone marrow-derived monocytes and are categorized into two main types, pro-inflammatory M1-type and pro-tumorigenic M2- type, which control tumor cells’ viability, proliferation, invasion, and angiogenesis [197]. A study on VS specimen showed a positive relationship between the expression of M2 macrophage marker CD163 and tumor growth and microvessel density. This finding indicates that M2-type macrophages in VS is associated with angiogenesis and volumetric tumor growth [198]. In another experimental study, de Vries et al. found that VS tissue had higher expression of macrophage colony-stimulating factor (M-CSF) and IL-34 (two cytokines regulating macrophage infiltration). In addition, the study demonstrated that the fast-growing VS and cystic tumors had significantly higher M-CSF expression. These findings address the positive link between M-CSF and VS progression [201]. In support, Lewis et al*.* in a composed imaging and neuropathology study, indicated that macrophages, rather than Schwann cells, are the dominant proliferating cells in the growing sporadic VS tumors [199].

The current understanding of the association between NF2-related VS and inflammation is limited to a handful of studies. Schulz et al*.* found that CD68^+^ macrophages are present in 90% [9/10] of NF2-related VS tumors, mainly with M2 phenotype [200]. Moreover, a higher expression of the macrophage marker CD68, T lymphocyte markers CD3 and CD8, and B lymphocytes marker CD20 was demonstrated in NF2-associated meningioma and schwannoma TME [202].

Another specified group of cells existing in the TME of NF2-related VS are myeloid-derived suppressor cells (MDSCs). These cells support tumor cells’ growth and survival by inhibiting the tumor-specific T cells [203]. Wang et al*.* demonstrated that MDSCs inhibit CD8^+^ T cells and induce their transformation into regulatory T cells by secreting TGF-β [203].

Detecting and quantifying the intratumoral inflammation biomarkers can provide new prospects for earlier detection and also allow specific targeted therapies. An in vivo study on nineteen VS patients with different degrees of growth (static, growing, and shrinking) aimed to find whether there is any difference between groups in terms of inflammation (using 11C-(R)-PK11195 PET scan) and vascular permeability (using dynamic contrast-enhanced MRI). The study showed that growing tumors (versus static tumors) had significantly increased inflammation and vascular permeability. The author introduced the 11C-(R)-PK11195 specific binding and DCE-MRI-derived parameters as imaging biomarkers of inflammation and vascular permeability in VS [199]. In a study of sporadic and NF2-related VS, Breun et al. showed the overexpression of C-X-C chemokine receptor type 4 (CXCR4) and also indicated the feasibility of CXCR4-directed PET/CT imaging of VS using the radiolabeled chemokine ligand [68 Ga]Pentixafor [204, 205].

A comparison between sporadic VS tissue and normal vestibular nerve showed a higher expression of pro-inflammatory cytokines IL-1β, IL-6, and TNF-α [206]. In alignment with these results, a study on secreted factors from human VS demonstrated higher levels of TNF-α secretion correlating with poorer hearing among patients and also can induce cellular loss in murine cochlear explants [70].

The nuclear factor kappa-B (NF-κB) is one of the main mediators of inflammation. NF-κB is a transcription factor participating in physiologic cellular processes like cell growth, apoptosis, inflammation as well as malignant transformation [197]. The inhibitory role of merlin on NF-κB has been proven in murine fibroblasts, rat glioma cells, and human schwannoma cell lines [207, 208]. Bioinformatic studies put forward NF-kB as a determining factor of VS pathogenesis [209]. To confirm this notion, Dilwali et al. examined whether selective inhibition of NF-kB (using siRNA and curcumin) has an inhibitory effect on the VS cells. The authors found that NF-kB inhibitors reduced VS cells’ proliferation and increased their death [209]. A computational drug repositioning platform to match known drug-gene interactions showed that mifepristone has the potential to treat VS [210]. Mifepristone is a progesterone and glucocorticoid receptor antagonist that can cross the blood–brain barrier. Since it is well tolerated when taken orally, it can also be utilized for the palliative benefits of glioblastoma [211]. Mifepristone can decrease VS cells' metabolic and proliferative activities and promote cytotoxicity in a dose-dependent manner, regardless of whether the *NF2* gene is mutated [210].Ingenuity Pathway Analysis showed that mifepristone targets NF-κB [210] as a pro-inflammatory transcription factor that participates in VS proliferation [209].

The *NLRP3* gene (NLR family pyrin domain containing 3) is a member of neuroinflammation-related signaling that mediates VS progression. The *NLRP3* gene product in a multi-protein complex triggers caspase-1 leading to the production of inflammatory cytokines, such as IL-1β and IL-18 [212]. So far, the mutated NLRP3 was reported as a reason for cochlear autoinflammation and syndromic and nonsyndromic hearing loss DFNA34. Anakinra, a nonglycosylated recombinant version of the human IL-1 receptor antagonist, reduced hearing loss in NLRP3 mutated patients [213]. Comprehensive pathway analysis using gene expression on VS microarray data together with validating this finding at the gene and protein expression level indicated higher expression of NLRP3 inflammasome in VS. Moreover, this overexpression is associated with reduced hearing loss in VS patients [214]. This finding suggests the therapeutic role of IL-1β blockade in patients with hearing loss secondary to VS.

The COX-2 (Cyclooxygenase-2) expression has been linked to VS proliferation in some studies [215, 216]. In NF2 patients, YAP activation (in the context of the Hippo pathway) enhances COX-2 production, which in turn catalyzes the prostaglandin E2 (PGE2) production. It has been demonstrated that PGE2 improves survival and proliferation and inhibits apoptosis in NF2-related schwannoma [184]. This finding shows that COX-2 inhibitors (such as aspirin) can diminish the progression of VS [217]. The inhibitory effect of aspirin on VS growth was demonstrated in a retrospective cohort. In this study on eighty-six patients, after an 11-year follow-up, aspirin significantly prevented VS growth (odds ratio [OR] 0.32, 95% CI 0.11–0.91) [218]. However, this study did not show a consistent correlation between VS growth and aspirin intake. Another two studies found no consistent correlation between aspirin administration and VS growth [219, 220]. Despite these controversies, aspirin is recommended by the Congress of Neurological Surgeons for VS patients [221]. To clarify this controversy, a phase II double-blind, randomized trial using aspirin on NF2-related or sporadic VS is underway (NCT03079999). Notably, the interaction between COX-2 and NF-κB pathway has been reported and aspirin modifies both NF-kB signaling and COX-2 expression [222].

A greater understanding of the inflammatory processes involved in NF2-related VS can assist in introducing novel targeted therapies.

### Immunotherapy

The last decades have witnessed a revolution in medical oncology with the development of immunotherapy. This approach aims to activate the immune system against tumor cells. Immunotherapy encompasses different modalities—including immune checkpoint inhibitors (ICIs), T-cell therapy, cancer vaccines, oncolytic virus therapy, and non-specific cytokines.

The first immunotherapy experience in NF2-related VS likely belongs to the study that applied VEGFR-specific cytotoxic T lymphocytes in patients with progressive tumors. This study demonstrated the safety and clinical efficacy of this approach [161]. A phase I/II clinical trial using antigen-specific T cells (CAR-T) and engineered immune effector cytotoxic T cells modified by immunoregulatory genes and immune-modified dendritic cell vaccine (DCvac) in the treatment of neurofibromatosis or schwannoma is underway [14].

Among all immunotherapy modalities, the most expansive body of research belongs to ICIs, especially anti-programmed cell death protein-1 (PD-1) or its ligand (PD-L1). These antibodies block the PD-1 and PD-L1 binding, thereby activating the immune cells against tumor cells [223]. Therefore, tumors with high expression of PD-L1 have higher responses to anti-PD-(L)1 antibody.

In addition, the type and rate of intratumoral lymphocyte infiltration are predictive factors of response to immunotherapies. Tumors with high infiltration of CD8^+^ T cells are good candidates for immunotherapy [224]. More responses can be expected if this condition coincides with high PD-L1 expression on tumor cells. The histopathologic examination of NF2-related VS reveals PD-L1 overexpression in up to 70–100% of the specimens [202]. This finding provides hope to apply anti-PD-(L)1 antibody for NF2-related VS.

Interestingly, PD-L1 expression may have a prognostic value in sporadic VS. In 2019, Perry et al. realized that PD-L1 expression on VS cells is associated with more tumor progression, poor facial nerve function, and poor tumor control. [225] This issue may be rooted in the association between inflammation and PD-L1 expression on tumor cells [226]. Dissecting the mechanisms behind the PD-L1 expression and their association with inflammation can be helpful for the future of immunotherapy in NF2-related VS.

Rutland et al. demonstrated a positive correlation between NF2 gene mutation and the extent of tumor-infiltrating lymphocytes in meningioma tissues. The authors demonstrated that NF2 mutations were present in 40% of meningiomas with 1–25 scattered lymphocytes and 54% of meningiomas that had even more scattered lymphocytes. On the other hand, NF2 mutation was not detected in meningiomas with no tumoral infiltration [227]. This finding put forward immunotherapies an interesting choice for treating NF2-related meningioma.

### Gene therapy

Gene-based therapies have provided novel opportunities for treating different diseases, such as retinal dystrophies, genetic hearing loss, and spinal muscular atrophy. In the case of retinal dystrophies, gene therapy has made a substantial contribution to therapeutics. In 2018, the FDA approved *RPE65* gene delivery by adeno-associated virus (AAV) for the treatment of Leber's congenital amaurosis [228].

Different research groups have focused on inner ear gene therapy methods, including viral and non-viral vectors, gene replacement, and gene suppression by RNA-based therapies and CRISPR/Cas9-based genome editing [229, 230].

However, when the outer and inner ear is targeted, gene therapy may become quite challenging due to the existing barriers and the question of how long viral vectors can continue their expression in target cells. In recent years, gene therapy implicated in hearing loss caused by genetic mutations has shown promising results. For instance, using the Anc80 vector, wild-type harmonin was effectively delivered into the inner ear of a mouse model of type I Usher syndrome caused by mutations in *Ush1c* gene encoding the protein harmonin. In addition to restoring mechanotransduction, the therapy led to remarkable improvements in complex audiovestibular functioning to approximately wild-type levels for a minimum of six months [231]. Another interesting case is the dual transduction of viral vectors containing otoferlin cDNA that led to partial recovery of hearing function and restoration of protein expression to 30% of levels in the wild-type in mouse models [232, 233].

The non-viral approaches are a potent alternate delivery treatment method that applies engineered non-viral delivery vehicles to satisfy the exact therapeutic need that is less immunogenic. In an in vivo study, delivery of cre-recombinase and genome editing agents through lipid compounds led to 90% recombination and 20% genome editing in newborn mouse OHCs-hair cells [234]. Furthermore, in a mouse model of dominant genetic hearing loss, injection into the cochlea of newborn mice reduced progressive hearing loss and increased hair cell survival in vivo. This was done by cationic lipid nanoparticles that incorporated Tmc1-targeting CRISPR/Cas-9 complexes [235]. Recently, tumor-penetrating nanocomplexes containing TNF-a short interfering RNAs (siRNA) were employed to target primary vestibular schwannoma cells in vitro actively; this method enables nanoparticles to be tumor tissue-specific with a systematic administration [236].

Several preclinical studies have indicated promising results with gene therapy in treating NF2. Merlin re-expression via gene replacement in NF2-null schwannomas led to increased apoptosis and tumor regression [237]. In a mouse model of schwannoma, direct injection of an AAV1 vector expressing caspase-1, under the control of Schwann-cell specific promoter, resulted in tumor regression [238]. In another study, AAV1 expressing ASC (Apoptosis-associated speck-like protein containing a CARD [caspase recruitment domain]) in human xenograft and murine allograft schwannoma models decreased tumor growth and resolved tumor-associated pain without detectable toxicity [239]. Peptide-based nanoparticles have been used to transport genetic materials to primary human VS cultures in vitro. This process was accomplished by coating the nanoparticle surface with a peptide that targets Schwann cells, reducing the release of an ototoxic inflammatory cytokine from tumor cells [240]. New evidence has put forward antisense therapy for the personalized therapy of NF2. In an in vitro study, antisense oligonucleotides targeting exon 11 rescued the NF2 phenotype [241]. It is still challenging to formulate gene or drug delivery methods that are effective for therapeutic purposes. Advances in the injection procedure through the round-window membrane will improve the injected medication's pharmacokinetics and pharmacodynamics within the inner ear [242].

## NF2 models in preclinical research

To date, the restricted availability of tumor tissues and, more importantly, the absence of in vitro and in vivo model systems are barriers to clinical studies of NF2-associated tumors and deciphering the biological mechanisms related to their progression [13, 243].

Currently, there is only one transformed and immortalized VS cell line harboring HPV E6 and E7 oncogenes (HEI-193), which due to long-term passaging, shows malignant and aggressive growth characteristics; therefore, they may not correctly depict the biology of NF2-associated tumors. This cell line serves as the basis for several NF2 drug-testing investigations [178, 244].

Because of the broad spectrum of clinical symptoms associated with NF2, reliable animal models that reflect the histology and biochemical variations seen in patients are more demanding.

While NF2 genetically engineered mouse (GEM) models are beneficial for studying the disease pathogenesis [30, 245], due to the prolonged timescales, intensive breeding requirements, and challenges in producing synchronized carcinogenesis, GEM models pose a challenge to conducting reliable drug testing [246].

Lately, patient-derived xenograft (PDX) models have emerged as an essential platform for evaluating novel pharmacotherapies; however, attempts to generate PDX for schwannoma have mainly unsuccessful.

A few studies have demonstrated the macroscopic development of transplanted human schwannoma tissue in naked mice. Nevertheless, these xenografts have limited effectiveness in preclinical research due to their slower growth and absence of transplantation capability [247, 248]. For instance, in two studies, HEI-93 cells were xenografted into the mouse sciatic nerve (one of them with luciferase activity), and both showed less accurate anatomical features compared to NF2 characteristics but had fast tumor development [64, 249]. Moreover, Periostin-Cre NF2flox/flox mice were shown to be authentic transgenic models of NF2 and VS but had complications in sustaining and obtaining synchronous tumors [245]. Furthermore, other models have shown no relevance to NF2 phenotypes despite their minimal advantages [249–252].

Interestingly, in a recent study, researchers developed PDX and cell lines resembling NF2 characteristics regarding histopathology, morphology and molecular biology. Their model was able to preserve patient *NF2* mutations, gene expression patterns mimicking patient tumor profiles, and many critical signaling pathways that are often dysregulated in human schwannomas [253].

New findings in human stem cell biology, organoid, and genome-editing techniques have provided the possibility to model nervous system tumors. However, we still face to lack of NF2 stem cell/organoid but models of inner ear organoids with full sensory circuits and myelinating Schwann cells raise new perspectives for paving the way for the development of the NF2 model [243]. Overall, establishing a reproducible experimental model of NF2-associated schwannoma has become a primary objective for the development of innovative and successful treatment methods, and there is a critical demand to fill this gap with further research.

## Conclusions

NF2 is a hereditary complex neuro-cutaneous disease with significant morbidities. Over the past decades, unprecedented steps have been taken toward a better understanding of NF2 pathophysiology. The researches have made significant progress, but some ambiguous issues still need to be clarified. For instance, despite widely accepted management options, like long-term monitoring, surgery, and radiation therapy, there is still no efficient treatment for NF2.

This review summarized the literature on NF2 regarding its clinical features, diagnostic criteria, genetic and epigenetic background, merlin biology function, and the available treatments and future perspectives. Applying the *NF2* gene as a target for gene therapy remains an open question. We believe that future studies can remove the veil of ignorance and answer some obscure aspects; for example, it is unclear how epigenetic modulations or modifier genes affect the variability in phenotype manifestation between individuals. We still need to learn more about how merlin participates in several intracellular pathways for the maintenance and proper function of cells or how the pathways manipulation can create new therapeutic perspectives for NF2 treatment. We believe that foreseeable investigations will shed light on merlin protein and its contribution to the disease process. This can, in turn, provide valuable information about its biological aspects, which will pave the way to utilize them effectively for therapeutic purposes.

## Data Availability

Not applicable.

## Abbreviations

NF2: Neurofibromatosis type 2
NF1: Neurofibromatosis type 1
SMARCB1: SWI/SNF related, matrix associated, actin dependent regulator of chromatin, subfamily B, member 1
LZTR1: Leucine zipper-like transcription regulator 1
VS: Vestibular schwannoma
NIH: National Institutes of Health
Schwannomatosis-NOS: Schwannomatosis not otherwise specified
Schwannomatosis NEC: Schwannomatosis not elsewhere classified
ERM: Ezrin/radixin/moesin
NGS: Next-generation sequencing
HOX: Homeobox genes
PI3K: Phosphoinositide-3 kinase
AKT: Ak strain transforming
Raf: Rapidly accelerated fibrosarcoma
MEK: Mitogen-activated protein kinase
ERK: Extracellular signal regulated kinase
RTK: Receptor tyrosine kinase signaling
mTOR: Mechanistic target of rapamycin
FERM: Band 4.1 ezrin radixin moesin
CTD: C- terminus domain
PTM: Post translational modifications
NLS: Nuclear localization sequence
PKA: Protein kinase A
PAK: P21 activated kinase
PDGFR: Platelet-derived growth factor receptor
EGFR: Epidermal growth factor receptor
HGFR: Hepatocyte growth factor receptor
VEGFR: Vascular endothelial growth factor receptor
PIKE-L: Phosphatidylinositol 3-kinase enhancer-L
GTP: Guanosine triphosphate
Lats1/2: Large tumor suppressor kinases
YAP: Yes-associated protein
TAZ: Transcriptional coactivator with PDZ-binding motif
TEAD: TEA domain transcription factor,
CRL4DCAF1: DDB1- and Cul4-associated factor 1
TCF/LEF: T-cell factor/lymphoid enhancer factor
LRP6: Low-density lipoprotein receptor-related proteins 6
Rac1: Ras-related C3 botulinum toxin substrate 1
JNK2: C-Jun N-terminal kinase
LIN28B: Lin-28 homolog B
Let-7: Lethal-7
HGF: Hepatocyte growth factor
PKD1: Phosphoinositide-dependent kinase-1
DCE-MRI: Dynamic contrast-enhanced magnetic resonance imaging
cMET: C-mesenchymal-epithelial transition
VGLL4: Mammalian vestigial-like 4
AMPK: AMP-dependent protein kinase
ALK: Anaplastic lymphoma kinase
TME: Tumor microenvironment
TAMs: Tumor-associated macrophages
M-CSF: Macrophage colony-stimulating factor
MDSCs: Myeloid-derived suppressor cells
CXCR4: C-X-C chemokine receptor type 4
NF-κB: Nuclear factor-kappa B
NLRP3: NLR family pyrin domain containing 3
ICIs: Immune checkpoint inhibitors
PD-1: Programmed cell death protein-1
PD-L1: Programmed cell death ligand siRNAs: short interfering RNAs
AAV: Adeno-associated virus
ASC: Apoptosis-associated speck-like protein containing a CARD
CARD: Caspase recruitment domain
GEM: Genetically engineered mouse
PDX: Patient-derived xenograft
